# “*You wished the ground would open and swallow you up”*: Expert opinions on shame, the collective, and other cultural considerations for suicide prevention among Asian American and Pacific Islander veterans

**DOI:** 10.1186/s40621-025-00560-6

**Published:** 2025-01-20

**Authors:** Evan R. Polzer, Carly M. Rohs, Christe’An D. Iglesias, Joseph Mignogna, Lauren S. Krishnamurti, Ryan Holliday, Lindsey L. Monteith

**Affiliations:** 1 VA Rocky Mountain Mental Illness Research, Education and Clinical Center for Suicide Prevention, Aurora, CO United States of America; 2 Department of Physical Medicine and Rehabilitation, University of Colorado Anschutz Medical Campus, Aurora, CO United States of America; 3 Department of Psychiatry, University of Colorado Anschutz Medical Campus, Aurora, CO United States of America

**Keywords:** Suicide, Veteran, Asian American, Pacific Islander, Culture

## Abstract

**Background:**

Rates of suicide remain elevated among U.S. Veterans and have increased disproportionately among Asian American and Pacific Islander (AAPI) Veterans. Knowledge is limited regarding suicide prevention considerations for clinicians working with AAPI Veterans, yet culturally responsive strategies tend to be most effective. To address this gap, we sought to elucidate subject matter experts’ perspectives regarding suicide prevention considerations for AAPI Veterans.

**Methods:**

Qualitative interviews were conducted with 14 key informants (e.g., clinicians, researchers) in 2023 to understand their experiences with, and recommendations for, preventing suicide among AAPI Veterans in the Continental U.S. Interview transcripts were analyzed through thematic analysis, with an inductive approach.

**Results:**

Key informants discussed the heterogeneity of the AAPI population and emphasized the need to balance cultural sensitivity and cultural humility in suicide prevention with AAPI Veterans. Fear of bringing shame and dishonor upon one’s family was described as a factor which may prevent AAPI Veterans from disclosing mental health concerns and suicide risk and which may prevent them from accessing healthcare services for mental health and suicidality. Suicide risk among AAPI Veterans was viewed as being shaped by shame and the centrality of the family-collective, with family conferring both protection against and risk for suicide. Cultural norms and beliefs regarding suicide were considered pertinent to suicide among AAPI Veterans and included beliefs about perseverance in coping with distress to permittance of suicide in specific circumstances. Somatic idioms were described as a means by which AAPI Veterans may communicate distress and suicidality, with key informants discussing how this may impact treatment and outreach.

**Conclusion:**

Key informant interviews provided crucial insights into cultural factors salient to conceptualizing and addressing AAPI Veterans’ risk for suicide. These findings can be utilized to inform tailored suicide prevention for this population, with emphasis on addressing mental health stigma, considering somatic idioms of distress, and considering the role of family in suicide risk and prevention.

**Supplementary Information:**

The online version contains supplementary material available at 10.1186/s40621-025-00560-6.

## Background

The suicide rate among U.S. Veterans nearly doubled from 2001 to 2021, resulting in a suicide rate in 2021 that was 71.8% times higher among Veterans than the suicide rate among non-Veteran adults [1]. While Veterans of nearly all racial and ethnic groups experienced increases in suicide rates over these two decades, Asian American and Pacific Islander (AAPI) Veterans experienced an unparalleled increase of 192.6%, with the rate increasing from 10.8 to 31.6 per 100,000 [2]. These findings are a clarion call to understand how to prevent suicide among AAPI Veterans.

A multitude of studies have focused on mental health and suicide risk among AAPI adults, highlighting important considerations for suicide prevention in this heterogenous population. For example, studies indicate that AAPI individuals may be reticent to disclose suicidal ideation [3] and to use mental health care [4, 5]. This may be due to several factors, such as mental health stigma within AAPI communities, where collectivism and filial piety (devoutness and duty to one’s family) may prevent disclosing psychological concerns [6]. Moreover, a lack of culturally competent care, cultural humility, and ethnically concordant providers may impede AAPI individuals from help-seeking for mental health concerns [7, 8]. The model minority myth regarding AAPI individuals has also been shown to be detrimental, resulting in AAPI individuals internalizing this myth and experiencing stress, pressure to succeed, and self-blame, while contributing to societal minimization and the invisibility of the AAPI community [9–12]. In addition to the aforementioned factors, recent events have prompted further concerns about suicide risk among the AAPI population. Specifically, AAPI individuals experienced psychosocial stressors during the COVID-19 pandemic, including a surge in AAPI discrimination, harassment, and violent hate crimes and harassment [13]. These factors have been associated with elevations in depression, anxiety, and suicidal thoughts and behavior [14, 15].

While these findings have been integral to advancing knowledge of suicide risk and prevention in the overall AAPI population, relatively few studies have focused on AAPI Veterans [16]. Nonetheless, studies to date have found that AAPI Veterans experience high rates of racism, particularly among those who served during the Vietnam War era [17], poorer mental health [18], and lower use of mental health services [19], although in one study, there was not an observed association between AAPI Veterans’ use of health services and perceived barriers and mental health stigma [20]. Specific to suicide, recent epidemiological studies have also revealed higher suicide rates among younger (ages 18–34) AAPI Veterans, relative to similarly aged Veterans in the overall population and older AAPI Veterans [21], as well as regional differences in suicide rates and methods among AAPI Veterans that appear to differ from the overall Veteran population [22]. A recent study reported substantial heterogeneity in the prevalence of suicidal ideation and suicide attempts among different ethnic groups of post-9/11 AAPI Veterans [23]. Additionally, suicide methods among AAPI Veterans differ in important ways, with lower use of firearms and heightened use of suffocation, further highlighting the need to elucidate idiographic suicide prevention considerations for this population.

Thus, further understanding of how to optimize suicide prevention for AAPI Veterans is requisite. The White House and Department of Veterans Affairs (VA) have advocated for tailoring suicide prevention approaches to be responsive to the unique needs and contexts experienced by different populations [1, 24]. However, the particularities of a tailored approach for AAPI Veterans remain unclear. One method to accelerate such knowledge is by interviewing individuals with knowledge and/or expertise relevant to preventing suicide among AAPI Veterans. Specifically, key informant interviews can facilitate understanding the unique needs of specific populations, in order to identify possible solutions and recommendations [25].

In this study, we aimed to elucidate key informants’ perspectives regarding considerations, needs, barriers, and facilitators for preventing suicide among AAPI Veterans in the Continental U.S, to elucidate culturally responsive considerations for preventing suicide in this population.

## Methods

Key informant interviews were conducted as part of a broader study focused on suicide prevention among AAPI Veterans in different U.S. regions (e.g., Continental U.S., Hawai’i, U.S. Pacific Island Territories) that included interviews with key informants and AAPI Veterans. The current manuscript focuses on qualitative interviews conducted with key informants in the Continental U.S. from January 2023 to December 2023. Project-specific Veteran engagement groups were formed for this project [26]; for this manuscript, Asian American Veterans with different lived experiences provided their input on study findings, resulting in greater depth and nuance to qualitative themes.

### Eligibility

For this analysis, key informants were operationalized as those with expertise relevant to suicide prevention with AAPI Veterans in the Continental U.S. Key informants could have expertise (i.e., knowledge and/or experience through clinical service delivery, research, and/or administration) with different aspects pertinent to suicide prevention, such as: mental health among AAPI Veterans (e.g., researchers in academic settings), administration or provision of mental health care or suicide prevention services to AAPI Veterans (e.g., suicide prevention coordinators), or mental health care and suicide prevention within AAPI communities (even if not specific to Veterans). Clinicians, researchers, community leaders, and administrators, within and outside the VA, were eligible to participate.

### Procedures

Key informants were identified through literature reviews, online searches, snowball sampling, and word-of-mouth. Professional listservs and social media were also used for recruitment. For interested individuals who were not initially invited to participate, a brief screening was conducted to confirm their study eligibility. Interested and eligible individuals were scheduled for a virtual one-hour appointment with study staff. The appointment commenced with informed consent, followed by a 1:1 qualitative interview, conducted by an experienced qualitative interviewer who used a semi-structured interview guide (developed by considering prior research and theory regarding this topic) to explore interviewees’s perspectives regarding AAPI Veterans’ experiences, as well as barriers, facilitators, needs, and recommendations for suicide prevention with this population (see Supplemental Appendix A). Interviews were, on average, 50 minutes in length. Qualitative interviews were audio-recorded and professionally transcribed. Participants were compensated $50 for participating, aside from those who were VA employees or who declined compensation. Study procedures were approved by the local Institutional Review Board and VA Research and Development Committee (COMIRB Protocol #21-4023).

### Participants

Fourteen individuals were consented and participated in this study. These key informants were identified by members of the research team (*n* = 10), suggested by invited or consented participants (*n* = 3), or recruited through a professional listserv email (*n* = 1). Participants’ relevant jobs/roles included the following (not mutually exclusive): psychologist (*n* = 5), VA leadership (*n* = 3), social worker (*n* = 2), VA suicide prevention coordinator (*n* = 2), professor (*n* = 2), Veteran service officer (*n* = 1), psychiatrist (*n* = 1), and epidemiologist (*n* = 1). Most (*n* = 10), but not all (*n* = 4), participants were affiliated with the VA. The majority of participants (*n* = 10) self-identified as AAPI. A few (*n* = 2) identified as Veterans.

### Analytic process

The study team included VA researchers and clinicians with expertise conducting qualitative research on Veteran suicide prevention, including with diverse populations. Backgrounds included clinical psychology, counseling psychology, public health, and anthropology. Two members of the research team identified as AAPI (one as Asian American; one as Pacific Islander), and all interviewers (JM, LK, LM, CI, CR) had training in qualitative data collection. Interviewers drafted analytic memos following each interview to note their initial impressions. Three trained qualitative analysts (EP, CR, and CI) reviewed and coded the interview transcripts. To facilitate objectivity, all analysts participated in a bracketing exercise to identify and discuss their personal preconceptions and potential biases prior to analysis [27]. Coders first familiarized themselves with the data by reading a subset of transcripts, then used an inductive approach to identify emerging themes and create an initial codebook [28]. The analysts used the interview guide to create a code hierarchy, which they subsequently piloted. The codebook hierarchy included: key informants’ experiences working with AAPI Veterans, and their perspectives on suicide prevention, considerations for clinical care, current interventions and programs, and recommendations for adapting these for AAPI Veterans. Once the final codebook was agreed upon, the remaining transcripts were divided between analysts. Each transcript was double coded. Qualitative analyses were performed using ATLAS.ti (v23.3.4.28863). Coders met routinely to reconcile differences in code application and usage, iteratively adapting the codebook as necessary based on emergent findings throughout the process.

## Results

### Summary of themes

Five themes were identified. First, key informants viewed the AAPI population as having immense diversity and heterogeneity, advocating for balancing cultural sensitivity with cultural humility. Second, shame was viewed as having a central role within AAPI cultures, being instilled throughout childhood and taking a more interpersonal and collectivistic form, while also deterring disclosure and help-seeking. Third, family was described as having a dual nature, with the potential to confer protection and risk against suicide. Fourth, cultural beliefs and tenets within AAPI cultures were considered relevant to understanding suicide, including concepts that promote perseverance in the face of hardship, as well as beliefs that may permit suicide in specific circumstances. Finally, key informants described how AAPI Veterans may communicate suicidality through somatic idioms, with implications for treatment and outreach.

### ***“There’s a great deal of diversity and similarity at the same time”***: **The complexity of AAPI as a label and the necessity of cultural humility**

The AAPI label was generally viewed positively for moving beyond simply referring to individuals as “Asian.” Nonetheless, key informants acknowledged the breadth of diversity among AAPI cultures, expressing concerns that, despite some similarities, the AAPI label can serve to disregard the variety of ethnic, cultural, and historical lineages subsumed within it. A social worker stated, “*I think when you hear the word Asian American*, *I mean that’s like very broad*, *right?…We’re not all the same. However*, *it’s interesting to see*, *I think*, *maybe just with some of the shared history*, *there are also a lot of similarities and kind of cultural values and norms. Despite the uniqueness of these populations*, *there’s also some similarities. So it’s like we’re different and the same all at once*.” Another key informant noted the limitations of the term in its breadth and lacking the specificity to address the diversity of groups categorized as AAPI. “*AAPI is a broad group*;” thus, it can be “*a bit of a misnomer to lump everyone together*, *because looking at all these different [cultural] factors makes it really hard to distinguish where large groups of people are coming from*.” An additional key informant noted the diversity of AAPI individuals, alongside the “invisibility” within society. *“I think for generations…it’s always been like*, *‘oh don’t worry about those Asian Americans*, *they’ll figure it out.’ And we don’t*, *not all of us…we’re not a monolith. Of all the monoliths*, *this is the least monolithic*, *right?… maybe it is out of the scope of y’all’s work*, *but just this idea of like*, *you matter…I think just societally and in general*, *Asian Americans are just*, *if you want to talk about an invisible group*, *you can’t point it more than Asian. Maybe outside of Native Americans.”*

Accordingly, key informants emphasized the need to balance knowledge of cultural differences among AAPI Veterans with an idiographic approach. “*Just because you’re an AAPI Veteran doesn’t mean that you’re going to think all the same. Everybody’s different.*” This key informant, who was a suicide prevention coordinator, also emphasized that providers should be cognizant of “*not having a certain set of assumptions or acting out on those assumptions when working with [AAPI Veterans]. They come from so many different cultures*.” Relatedly, a social worker stated: “*I think for people doing this work with this population*, *I think taking the time to learn more about the culture that this Veteran is coming from. So doing your own study*, *informing yourself. And then*, *of course*, *asking Veterans. We’re not a homogenous group. So you’re going to have to ask them about their experience within that culture*, *how they experience it. So kind of asking Veterans questions about their cultural experiences*, *as well as learning more about that culture, is important. We need to have culturally informed providers*.” Thus, key informants emphasized the importance of taking an approach to suicide prevention with AAPI Veterans that includes both cultural sensitivity and cultural humility. “*I think having cultural humility and sensitivity for providers*, *for educators*, *for people who are working in suicide prevention is what’s needed in order to ensure that messaging* ,*treatment*, *whatever*, *is accessible and non-stigmatizing and offered in a sensitive way and engages everyone equitably*,” an epidemiologist described.

Relatedly, a few key informants noted that some AAPI Veterans may prefer to work with an AAPI provider, who may more readily understand their experiences. For example, a psychologist stated: “*My experience has been that oftentimes AAPI Veterans would like to work with an AAPI therapist*, *and that’s just obviously not always possible*, *but I think it’s noteworthy*, *and I can appreciate and respect their preference for having that. There’s just less explaining*, *I guess*, *that needs to be done a lot of the time…there’s certain terminologies*, *certain tropes*, *certain culture-specific paradigms even or experiences that a person who is not in that group may not understand. And they may be very well capable of understanding*, *but the individual doesn’t always want to have to explain that every time.*”

### ***“The collective is both the source of support and the source of despair”***: **Shame**,** stigma**,** and the primacy of the family-collective**

Key informants suggested that suicide risk among AAPI Veterans should be viewed as reflective of the broader cultural milieu that shapes the mental health processes leading to suicide. They described how varying cultural practices, beliefs, and norms during one’s upbringing shape how mental health and suicide are understood, communicated, or not communicated– for example, in preventing the disclosure of mental health concerns and suicidality among AAPI individuals.

Specifically, shame was one of the most common and powerful factors described as influencing the mental health of AAPI Veterans. Key informants commonly described AAPI cultures as “shame-based” or as “shame societies.” A psychiatrist stated, “*Asian cultures are shame-based cultures*, *where shame is a big part of the culture*.” Shame was described as manifesting both through an interpersonal and communal form and as a negative, internalized emotion experienced by the individual. “*Shame can manifest itself in many ways*, *but where it gets more culturally salient for Asian Americans is in a couple of areas where it’s more interpersonal in nature*,” a psychiatrist stated. Key informants routinely emphasized that in many AAPI cultures, the collective takes precedence over the individual, and shame is perceived as reflecting not just upon one’s own perceived failures or shortcomings, but also reflects upon one’s family and culture. A psychologist detailed how “*Asian American cultures are very collectivistic*, *you really think of the role that you have in the broader culture*,* the family unit…it’s not really isolated to yourself*.” Another psychologist described a similar sentiment: “*There’s a collectivistic nature in AAPI cultures. One person represents the group. So if somebody has some mental health issue*, *it also doesn’t represent the group well*. *That can be seen as it negatively impacts the group as a whole*.” In this sense, key informants expressed that shame is amplified and may have broader effects in that the individual also experiences the shame of failing their family and culture. Another psychologist described how the collectivism within AAPI cultures differs from the individualism within Western culture and may prevent help-seeking: *“So there’s like the aspect of community and what’s good for the group versus the way that we are very individualistic in Western culture*,* particularly in the U.S. I think that maybe that could be somewhat related in preventing people from thinking that maybe their needs are important enough or significant enough to go seek help or resources.*”

Key informants described how shame is used early in childhood in some AAPI cultures. “*Shame is used as a method of parental socialization in many Asian cultures*,” a psychiatrist explained: “*The way you socialize a kid is to shame them. You say ‘Hey*,* if you do this*,* you’re gonna embarrass our family. You’re gonna bring shame to yourself and your family. You’re gonna make us look really bad*.’” Many key informants discussed authoritarian parenting styles, with some mentioning “tiger moms” in some AAPI cultures. Key informants explained how this form of parenting–strict, demanding, and with high expectations, often at the expense of the child’s social or emotional well-being–instrumentalizes shame as a motivator, pushing individuals towards educational, financial, and professional achievement, with the fear of failing to live up to these standards developed in childhood. A key informant noted that this type of parenting may arise from the fact that “*a lot of [AAPI] immigrants had to come to the U.S. with very little*, *and so their parents had to sacrifice and work hard for their families*.” A psychologist described this form of parenting as utilizing shame to instill “*perfectionism*, *it’s just constant striving and constant improvement and just a very hard work ethic*.” A suicide prevention coordinator discussed how an AAPI Veteran client had described his parent as being “*very tough emotionally on him*, *even as a little child*.” A psychiatrist described working with Veterans who grew up with “tiger moms,” noting how children were “*expected to suck it up and move on and not show weakness. The only feelings that were acceptable were anger*.” Connecting this form of cultural upbringing with military service and culture, a suicide prevention coordinator noted how, for AAPI Veterans, it is a “*seamless transition from family to that same kind of culture where you’re not to show feelings and you are just kind of expected to succeed at your own expense*.”

Key informants noted the potential issues that shame can have on AAPI individuals’ mental health. “*This notion of shame*, *of bringing shame to the family or bringing shame to the family name*, *I think if someone is struggling with shame for whatever reason*, *thinking they haven’t been a dutiful child*…*or they didn’t meet some type of societal expectation*, *I think that could lead to certain thoughts around shame*, *guilt*, *hopelessness*,” a social worker stated.

Key informants also indicated that shame may heighten stigma regarding mental health issues, which was noted to be common in many AAPI cultures, and that this stigma may deter individuals from seeking help for mental health concerns. “*In a lot of [AAPI Veterans’] communities*, *there’s still stigma around mental health. It’s very taboo. If you talk about going to see a psychiatrist*, *it’s seen as shameful even among some family. It’s not discussed. It’s not talked about openly. There’s even like this idea*, *okay*, *we can’t let this information come out*, *right*, *because it’ll bring shame to the family*,” a social worker expressed. Key informants noted that mental health care is stigmatized and viewed as shameful in some AAPI cultures due to mental health concerns being perceived as a potential threat to group cohesion and harmony, both of which are culturally valued. “*The cultural stigma against seeking help would be a major barrier or risk factor for suicide*,” a psychologist stated. “*They’re less encouraged to reach out for help when they’re experiencing distress*, *particularly suicidal thoughts*.” Key informants indicated that concerns about confidentiality can further impede AAPI individuals from seeking out mental health services, due to the fear that their family name might be disclosed to the community and that this may further exacerbate their risk for suicide. As one psychologist expressed, “*I think feelings of shame*, *feelings of being alone*, *feeling like you’re a burden on others*, *that disclosure would be very shameful. I think oftentimes even the immediate family isn’t aware of things that are going on and so that othering-ness in all your circles*, *including your most immediate circle*, *can be very isolating and lead to further suicidality*.”

### ***“Look how important this family is to me***, ***that I would be willing to die for the family”***: **Shame**, **dishonor**, **and the dual nature of family**

Family was seen as a double-edged sword that could positively or negatively influence suicidality among AAPI Veterans. “*Sometimes family can be both a vulnerability and strength at the same time*,” a social worker stated. Discussing the dual nature of family in AAPI individuals’ suicide risk, a psychologist stated: “*I would say that like a lot of times for safety planning*, *people would say like*, *‘My family is a source of strength and support’*, *and that’s a reason to not do it…There tends to be a lot more conflict around the role of family. It’s like*, ‘*The pressure from the family leads me to want to do this*,* but I can never disappoint them. I can never hurt them by following through with it.*’”.

Thus, in one sense, family was perceived as conferring protective benefits, such as by acting as a powerful reason for living. “*Even if they are fighting*, *they still want to be there for the children. They want to watch them grow up…just to be there for them as they get older*,” a psychologist stated. Key informants discussed how the cultural emphasis on family within AAPI cultures may deter some AAPI Veterans from attempting suicide. A psychologist wondered if this could be used clinically with AAPI Veterans to prevent suicide: “*There’s a little bit of ambivalence because they knew they were hurting their family members. If we can play out that love with those family members a little bit more*, *could we have helped prevent them from taking their last steps?*” Key informants also noted that family can be protective to AAPI Veterans by providing them with support when they are suicidal. “*[In] Asian cultural values*, *if you come from a family that’s very family-oriented*, *maybe you’ll be able to get support from that*,” a social worker stated. Similarly, a psychologist noted how family support could be particularly protective for AAPI individuals due to the collectivistic nature of AAPI cultures: “*I think having a community*,* whether that’s family or friends*, *having that strong social support [is protective] because AAPI countries are more collectivistic*.” Another psychologist emphasized how familial belonging could be protective against suicide, “*I think as far as like belongingness*,* the fact that it’s more of a collectivistic culture and that there’s usually a lot of ties to family and extended family and community*,* I would say that that aspect is protective.*”

Key informants noted, however, that the heightened emphasis on family could have a particularly detrimental effect on AAPI Veterans’ suicide risk when familial stress occurred. A social worker noted, “*That is something I’ve noticed in working with this population is seeing a lot of themes that are very similar*, *like strong filial ties…the sense of duty towards parents…close family ties and then impact of that on one’s mental health and their view of self when there’s some disruption or dysfunction or trauma within the family.*”

Key informants also described how the heightened emphasis on family within AAPI cultures could further exacerbate AAPI Veterans’ risk for suicide by preventing disclosure and help-seeking. In particular, they noted pressure among AAPI Veterans to protect their families from shame and embarrassment and to “*save face*.” A Veteran service officer described, “*You don’t make your family look bad. Meaning you don’t air out your dirty laundry*, *or you don’t make any news that may be portrayed negatively*, *like having a mental health issue*, *or having a mental breakdown*.” Key informants discussed AAPI Veterans’ concerns about bringing shame to their families if they disclosed negative emotions, distress, mental health concerns, or suicidality. “*There’s so much stigma and pressure in disclosing and sharing your emotional pains because it might bring dishonor to the family or taint the community perspective of your family*,” a psychologist expressed. A psychologist described how this may lead AAPI Veterans to suppress their emotions, noting that there is “*an encouragement of suppression of emotions*, *rather than expression of them…It could be shameful for families if they had a family member who was suicidal. Maybe that person wouldn’t be as likely to reach out for help because they’re afraid of shaming their family*.” Thus, key informants noted how this pressure to avoid embarrassing or shaming one’s family may prevent AAPI Veterans from seeking care when they are suicidal or experiencing mental health concerns.

Some key informants described ways in which these familial concerns could potentially be addressed. For example, a psychologist described working with AAPI clients to identify non-familial sources of support: “*I try to help them find other sources of support*, *maybe outside of their given family. If they have other chosen family or other people who they can call to have support around. But it’s really*, *really hard because there is the shame around it*.” Another psychologist described the importance of how therapists discuss familial issues with AAPI clients:

*Just from my understanding of therapy with Asian Americans*, *often the challenge comes in some kind of a conflict with family members and the parents*, *but without the desire to completely separate from one’s family and parents. And so it’s kind of a Western notion of finding your true self free from people’s expectations is how we work. And Asian American clients will sometimes talk about that*,* that ‘my therapist was almost trying to get me to hate my parents’ because they would share all these horrific things that their parents did and*, *of course*, *their therapist who is very empathic would say things like*, *‘I can’t believe your parents did that to you.’ And then they take it as an attack on their family—and then they feel shame about it. So this is where a lack of cultural understanding of how complex this sense of deep ambivalence about one’s family where you feel a sense of pain when it got to your family members*,* but you also feel loyal to them. That ambivalence is something non-Asian American therapists may not fully grasp…what I’ve done is to assure that*, *in these types of cultural conflicts*, *to identify the source of the problem as more structural in nature rather than interpersonal. Rather than locate the source of the problem as your parents…just say that*, *‘you know*, *what I’m seeing here is that you and your immigrant parents are the product of two different cultural systems…and the problem lies not in you or your parents*, *but in the way you have been socialized differently*, *in different cultures’…that sort of takes away the blame from individuals to a more societal issue.*

### **“*Suicide is seen as an honorable escape if you tried so hard to do all the things that are asked of you***,*** and you really cannot reach it”***: **Cultural allowances for suicide and resiliencies**

Some key informants spoke of there being greater cultural allowances for suicidal acts in specific circumstances within some Asian cultures. “*If a person’s culture of origin or family’s culture of origin has particular elements in which suicide is not some anathema or even taboo concept*, *that might also lead the person to having maybe just like a more nuanced look on it*,” *a* suicide prevention coordinator stated. A psychologist noted that, rather than bringing shame upon oneself and one’s family, suicide could be viewed as a more honorable or respectable option in extreme circumstances in some Asian cultures, such as among Japanese, Korean, or Chinese cultures. “*There’s a lot about suicide in our culture that is very*, *almost like revered or seen as honorable.*” She noted: “*Suicide is seen as a possible out more so in Asian countries than Western cultures*. *I wouldn’t say it’s condoned*, *but it’s not like the worst thing possible the way that like Christianity*, *like the Western culture that’s rooted in like Anglo-Saxon Christian values*, *where it’s like'oh*, *you’re gonna go to hell if you die by suicide.’*” She further elaborated: “*But I think culturally when it comes to suicide prevention*, *this is true for several Asian cultures*, *I’m thinking specifically for East Asian cultures*, *Japan*, *China*, *and South Korea*, *there is almost like an honorable escape. Suicide is seen as an honorable escape if you tried so hard to do all the things that are asked of you*, *and you really cannot reach it…suicide is seen as a possible out more so in Asian countries than Western cultures.*”

Conversely, several key informants discussed cultural concepts in AAPI cultures that promote perseverance, persistence, and inner determination in the face of hardship, noting that these may be protective to some AAPI Veterans when experiencing distress or suicidality. For example, as a psychologist pointed out, some Eastern philosophies, such as Buddhism and Taoism, teach that “*suffering is a part of life…There’s all these different philosophies that can be very healing and accepting that can help somebody cope with their suicidal thoughts or depressive symptoms*.” Expanding on this, a psychologist noted that while “*there are a number of things that are considered new in the Western world*,” with therapeutic modalities like cognitive behavioral therapy and acceptance and commitment therapy being “*definitely more of a Western approach*,” mindfulness and acceptance of life’s hardships “*have roots in ancient Eastern philosophies.*” Additionally, key informants noted that some AAPI cultures have unique cultural or linguistic terms, utterances, or beliefs that confer protection to individuals experiencing challenges. For example, a key informant noted how Japanese concepts like *gaman* (“endure”) and *shikata ga nai* (“it cannot be helped”) may serve as coping statements to strengthen resolve and promote resiliency.

### ***“If you don’t have the language for emotional expression of pain***,*** it comes out physically”***: **Somatic idioms of distress and treatment considerations**

Given the cultural stigma and shame associated with expressing individual negative emotions, mental health concerns, or suicidality, key informants noted that AAPI Veterans may experience and communicate distress differently, such as by discussing somatic symptoms or complaints. “*Like you could say oh*, *you have a headache or gastrointestinal problems. Like that’s more acceptable than saying ‘hey*, *I’m not getting along with my wife' or something like that*,” a psychologist stated. Another psychologist described how AAPI Veterans may communicate through vague or somatic terms: “‘*I don’t feel good. I’m tired all the time. I don’t feel like doing anything.’ I think that they’re gonna describe things in a lot more somatic terms. It’ll kind of show as something medical*, *rather than mental health*.” Thus, key informants indicated that, rather than expressing suicidality outwardly, AAPI Veterans may use somatic or bodily idioms to describe their distress. “*I think that the warning signs can also be very different*,” a psychologist stated. “*Oftentimes Asians don’t necessarily have that language or feel comfortable with it. There can be warning signs that are physical*, *things that are happening in the body*.” A psychologist described how AAPI individuals may use somatic idioms “*because of the stigma associated with talking and also the way that distress is felt*, *culturally*, *in terms of more bodily experiences*, *maybe things that they don’t necessarily have the words to put into*.”

Some key informants noted that this may result in AAPI individuals being referred for mental health care by medical professionals who have ruled out medical explanations for their symptoms, despite these being their presenting concerns. A psychologist noted “*the amount of somatic complaints that show up*, *especially for first generation or like recent immigrants.*” This key informant elaborated, “*A lot of times they come to me because they saw their GI*, *their eye doctor because their vision was going blurry or grayscale*, *or they were throwing up every single day or having these terrible migraines*, *because…if you don’t have the language for emotional expression of pain*, *it comes out physically. And so they go to a cardiologist*, *and they’re like ‘your heart is actually fine*,’ *and they recommend them to see me because they’re actually having panic. It’s like the layers. It shows up differently because it’s such a somatic manifestation*, *and they don’t have that awareness of it yet.*”

Thus, key informants emphasized that these idioms of distress, as well as potential warning signs of suicide, may not be readily understood or recognized by providers as such. For example, a somatic idiom (e.g., being “*tired all the time*”) could be interpreted to mean a generalized lack of physical energy, rather than as potentially being indicative of more serious psychological distress. Accordingly, key informants noted the importance of increasing providers’ awareness that AAPI Veterans may use these idioms to convey distress. A psychologist also emphasized the importance of “*training them in the assessment of nonverbals a little bit more and like reading into what the patient’s saying*, *that the symptoms they’re expressing could be other things. So*,* okay*, *this symptom could be hypothyroidism or whatever*, *but it could also be depression. And just kind of opening that up in their mind that*, *okay*, *this could also be something mental health related that the patient isn’t aware of and isn’t able to express. And it’s kind of the professional’s job to identify that or help to identify it. So kind of training in like listening to what the patient’s saying*, *but also understanding that it might not be as straightforward as what they’re coming and presenting with. There could be a whole lot under that that needs to be assessed.*”

Additionally, key informants noted how cultural differences in the experience, perception, and communication of distress could impact treatment. “*I would also say that the way that distress is perceived in Asian American individuals may also be different than the way that it is typically talked about in more traditional psychotherapeutic treatments*, *or evidence-based treatments that are part of the VA system*,” a psychologist noted. Another psychologist stated regarding AAPI cultures: “*There’s a lot more normalization in talking about somatic complaints and the ways that mental health can manifest physically. But on the other hand*, *there’s a lot more*, *kind of a holistic perspective when it comes to wellness. Mental and physical health are not really seen as separate entities*, *so the focus does tend to be more on the physical*, *and there’s less emotional vocabulary or normalization of talking about experiences and emotions.*” Also noting these differences in spoken language and presentations of distress, a psychologist explained how “*some of these treatments kind of assume that people have a baseline of being able to talk about those things*, *and so individuals are already coming at a deficit when they’re starting treatment*.” A psychologist described how these factors may impact AAPI individuals’ preferences for, and receptivity to, different types of treatments. “*At least in some parts*, *some AAPI countries*, *like the Chinese*, *there’s a long history of Chinese medicine and so they just view healthcare and health problems differently*, *a balance of some type in the body and so it’s not something you would seek Western therapy for. For example*, *you might take Chinese medicine for some mental ailment versus going to a therapist to talk about your problems*, *you know*, *talk therapy. Or even taking antipsychotics or antidepressants*, *there may be some resistance beyond the stigma. It’s just the medicinal*, *different medicinal approach.*”

While key informants noted aspects of psychotherapy, such as naming and discussing one’s emotions, that may be inconsistent with some AAPI cultures, some key informants described ways in which the emphasis on somatic experiences could be incorporated into treatment to be more culturally responsive. For example, a psychologist noted regarding their approach to working with AAPI Veterans, “*I try to take a very multicultural approach*, *so thinking a little bit outside the box. So it’s not just about like okay*, *I’ll make a referral to psychiatry and for psychotherapy*,* but it also might be*, *is there*, *like some people might be looking to get reconnected with a spiritual set of resources. Is there an acupuncturist*, *for example*, *or somebody who is well-versed in Chinese herbs and medicines? Eastern medicine even that you can go to*, *to supplement the more traditional treatment modalities that we offer. Things like that.*” This key informant wondered about the potential to use different approaches with AAPI Veterans to integrate or complement treatments: *“There’s all these alternative medicines that the AAPI communities use…for example*, *Chinese medicine is used by many AAPI cultures and not just Chinese Veterans*, *other AAPI Veterans*, *they use Chinese medicine and Chinese herbs…and there’s a whole bunch of other approaches*, *acupressure….so VA could maybe think about how they could synergize different approaches.*”

Key informants also noted how these differences could translate to targeting messaging and outreach to AAPI Veterans in medical settings or in Primary Care - Mental Health Integration (PC-MHI) settings. For example, a psychologist stated, “*I would like target the more medical departments because I don’t think Asians are likely to use a mental health clinic as their entry point. I think they’re more likely to go to primary care…they’ll seek medical care over mental health care first. So advertising there or providing resources in medical clinics would be a great idea…PC-MHI is great because there’s like mental health staff right in primary care*, *so that would be like a clinical resource for both the Veterans and the staff. But also like information*, *like flyers*, *posters*, *things given out at the visits*, *like brochures of educating people about the mental health resources in the clinic available.*”

## Discussion

There has been a call to tailor suicide prevention strategies for the needs and contexts of different populations [24], as research suggests that interventions are more effective when adapted for different populations [29]. Our findings from interviews with key informants highlight several important considerations for suicide prevention with AAPI Veterans in the Continental U.S.

Consistent with prior findings in which shame was central to AAPI cultures [30, 31], shame was considered highly salient to suicide risk among AAPI Veterans, reflecting the importance of the family-collective within AAPI cultures. Moreover, the interplay between shame, stigma, and the family-collective was perceived as impacting risk and protective factors for suicidal thoughts and behaviors. Numerous published studies have described the sociological function of shame generally and cross-culturally [32, 33], as well as its impact on mental health [34, 35]. Our findings also align with previously published work that describe efforts to “save face” [36] and protect one’s family from shame and dishonor within many AAPI cultures [37]. This can inculcate mental health stigma and prevent distressed individuals from obtaining healthcare [38]. Indeed, mental healthcare stigma has been noted for AAPI populations [39, 40] and was noted by key informants within the present study. While prior work has focused on sources of shame that service members and Veterans experience during their military service [41, 42], and how these may impact service utilization after separation [43], little has been done to understand how overlapping, concurrent models of shame influence mental health and help-seeking.

Further evidence suggests decreased openness to mental health care among AAPI Veterans relative to other racial and ethnic groups [44, 45]. The current study reinforces the need to destigmatize discussions of mental health and help-seeking among AAPI Veterans, such as through public health campaigns tailored to AAPI Veterans to attenuate shame and internalized stigma [46]. Such strategies may be more effective if they include representation of AAPI Veterans in materials or include local vernacular, particularly considering the historic “invisibility” of AAPI individuals [47]. Another strategy, as suggested by a key informant, is focusing messaging and outreach efforts for AAPI Veterans in primary care or medical settings, which may ameliorate some of the stigma of seeking care in mental health settings [48]. Nonetheless, further research is needed to understand how best to tailor messaging, materials, and outreach to AAPI Veterans.

Another important finding concerned the centrality of family, which could function as a protective factor (e.g., as a reason for living and source of support) and risk factor (e.g., by deterring disclosure of suicidality and help-seeking). Perceived social supports, including family, can confer numerous protective benefits for individuals experiencing distress [49]. Moreover, cultural norms that prioritize family, as well as language barriers, discriminatory practices, lack of ethnically concordant providers [7], and institutional obstacles may result in AAPI individuals relying upon family for support in lieu of seeking mental healthcare [50]. However, the centrality of family as the predominant source of support [51] can have negative impacts. AAPI individuals may be reticent to turn to family for support when experiencing distress, as numerous cultural values emphasize the need to avoid bringing disharmony, shame, or imbalance to the family unit [52, 53]. This can result in AAPI individuals exercising greater self-concealment of mental health concerns [54, 55] and result in numerous negative impacts on mental health and wellness [7, 56]. Furthermore, familial strain may also prevent individuals from turning to family for support, resulting in increased loneliness and negatively impacting mental health [57, 58]. Consequently, it is important to consider these factors when conducting interventions with AAPI Veterans that involve family. For example, in safety planning, providers can work collaboratively with Veterans to explore their beliefs and willingness to reach out to family for distraction or coping to determine the feasibility and utility of their inclusion as supports.

In addition, key informants described distinct cultural beliefs and practices that may be important to consider in augmenting suicide prevention. For instance, numerous key informants noted varying Eastern spiritual, philosophical, and meditative tenets that have long been seen as conferring productive mentalities. Importantly, several empirically-supported treatments (e.g., Dialectical Behavior Therapy; Mindfulness-Based Cognitive Therapy) include elements of mindfulness and acceptance; nonetheless, the efficacy of some of these treatments in addressing suicide risk among AAPI Veterans remains understudied. Moreover, understanding how best to integrate philosophical and meditative tenets into acute suicide prevention interventions (e.g., safety planning) requires further understanding. Finally, given the inherent diversity of the AAPI population, including with respect to the prevalence of suicidal ideation and suicide attempt [23, 59, 60], the present findings may not apply to all AAPI Veterans, who may not uniformly ascribe to all of the aforementioned beliefs and practices. As such, providers should conduct an idiographic assessment of a Veteran’s cultural background and beliefs as part of a comprehensive biopsychosocial assessment to understand the role of culture in clinical conceptualization, in order to inform treatment planning and intervention.

Key informants also described cultural allowances in some AAPI cultures that may mitigate some of the taboos surrounding suicide. Consistent with sociocultural theories of suicide [61, 62], and the cultural theory of suicide [63], the present findings highlight the ways in which suicide is moderated by an individual’s cultural milieu, with culture outlining the means, conditions, and responses for which suicidal behaviors are culturally sanctioned [64–66]. While not reflective of all AAPI cultures or individuals, awareness of the sociocultural dimensions of suicide, including how suicidal behaviors are perceived within different AAPI cultures, can facilitate cultural sensitivity and awareness for a more holistic understanding of cultural factors that may increase risk for suicide among AAPI Veterans.

Similarly, key informants described potential differences in how AAPI Veterans express distress and suicidality. In contrast to the more psychological or cognitive expressions of suicidality commonly identified in Western cultures, AAPI Veterans may use somatic descriptors [67]. This closely aligns with recent empirical literature detailing how the somatization of suicidal distress may be seen as a form of “hidden suicidal ideation” among AAPI individuals [3, 68]. Anthropological and psychological research discussing these “idioms of distress” highlight how they provide individuals with an alternative means of denoting distress [69, 70]. AAPI individuals may use somatic idioms to describe their distress due to cultural differences in mind-body dualism (as seen in Confucian and other Eastern philosophies) or to avoid stigma associated with psychological distress and help-seeking. Provider awareness of different idioms of distress (e.g., chest pain, lack of energy, chronic weariness [71]) among AAPI Veterans may facilitate greater detection of suicide risk and working collaboratively to address risk. Further study of existing, or development of new, measures for assessing suicide risk that account for somatic complaints that could indicate psychological distress among AAPI Veterans is warranted.

### Limitations

The ability to interview subject matter experts, who offered invaluable insights and experiences, is a strength, but also a potential limitation. While many of the key informants also identified as AAPI, few were Veterans, and it is possible that AAPI Veterans may have different perspectives. However, a project-specific Veteran engagement group, which included Asian American Veterans with different lived experiences, did provide input on findings. Additionally, forthcoming work is examining AAPI Veterans’ lived experiences and perspectives regarding suicide risk and prevention. In addition, while efforts were made to elicit perspectives from a range of individuals with expertise relevant to AAPI Veteran suicide prevention, the higher proportion of psychologists (and lower proportion of social workers, psychiatrists, and professionals from other disciplines) within the sample may have skewed findings. Furthermore, key informants had different levels of experience working directly with AAPI Veterans at risk for suicide. Additionally, despite bracketing and similar exercises aiming to mitigate potential biases amongst study team members, the composition of the research team included some individuals from AAPI backgrounds but no Veterans. This could have influenced data collection and interpretation, such as inadvertently resulting in some perspectives and considerations receiving greater perceived import and detail. Finally, the interview guide focused broadly on AAPI Veterans, yet disaggregation between Asian American and Pacific Islander Veterans or by ethnicity is optimal given the heterogeneity of AAPI cultures.

## Conclusion

Preventing suicide requires understanding not just the factors contributing to suicidal thoughts and behaviors, but also how to tailor suicide prevention initiatives to meet the needs and contexts within different groups. By interviewing subject matter experts, this study provides new knowledge regarding cultural considerations to preventing suicide among AAPI Veterans, a pivotal first step to engaging AAPI Veterans in culturally responsive suicide prevention.

## Supplementary Information

Supplementary Material 1

## Data Availability

Data utilized in this study cannot be publicly shared because of privacy and confidentiality requirements. De-identified data may be available from the VA R&D Committee (phone contact: 303-399-8020) for researchers who meet the criteria to access confidential study data.
