# Supplementary material for: “You wished the ground would open and swallow you up”: Expert opinions on shame, the collective, and other cultural considerations for suicide prevention among Asian American and Pacific Islander veterans
Source: Inj Epidemiol. 2025 Jan 20;12:5. doi: 10.1186/s40621-025-00560-6 (PMC11749089; doi:10.1186/s40621-025-00560-6)
Supplement: Supplementary file 1 — Supplementary Material 1 [file 40621_2025_560_MOESM1_ESM.docx]

**Aim 1 Interview Guide**

*As is conventional for qualitative research using semi-structured interviews for data collection, the exact wording of questions and probes will vary. The questions and probes listed here are representative of the general content to be explored in the interview. Not every question may be asked of every participant depending on interview flow and timing.*

***NOTE: Content in brackets will be modified, depending on the relevance to the individual being interviewed. Probe throughout and modify wording based on whether the participant is providing expertise regarding Asian American (AA) Veterans, Pacific Islander (PI) Veterans, or both.***

Thank you for your willingness to participate in this interview. As a reminder, there are no right or wrong answers to any of these questions. Interviews are voluntary and confidential. While anonymity is not possible, anonymity will be protected such that the research team will keep the identity of respondents confidential, and identifiers will also be kept separate from the coded data. Results will be reported in an anonymous format in reports. All quotes will be de-identified when transcribed or published. **Do I have your permission to begin audio-recording?**

1. **How did you find out about this research study? What motivated you to participate?**
2. **What comes to mind when you think of AAPI Veterans? How do you personally define AAPI?** *[Clarify focus of this interview regarding the subsequent questions]*
3. **Please describe your current job/role.**
4. **Please tell me about your experiences working with AAPI Veterans. [Probe whether they have worked with AA Veterans, PI Veterans, or both.]**
   1. When did you begin working with AAPI Veterans? What was that like for you?
   2. What other experiences have you had working with AAPI Veterans?
   3. Do you work with AAPI Veterans in your current position? If so, what does this entail?
   4. Can you tell me more about the specific AAPI populations you have worked with? What has that been like for you?
   5. In what types of settings have you worked with AAPI Veterans? In which regions have these Veterans resided? Have your experiences working with AAPI Veterans varied based on the geographical location or region you were in? If so, please describe.
   6. [For interviewees who are researchers] Please tell me about research you have conducted related to AAPI Veterans.
   7. [For interviewees who are clinicians] Please tell me about the clinical care you have provided to AAPI Veterans. What percentage of your patients are AA or PI Veterans?
      1. What trainings or resources have helped you address the specific needs of these groups?
5. **Do you think that AAPI Veterans’ experiences before, during, and after their military service differ, compared to Veterans of other racial or ethnic backgrounds? If so, please describe.**
   1. Are there any experiences that are unique to specific AA or PI Veteran communities? For example:
      1. How, if at all, do experiences of Asian American Veterans differ from those of Pacific Islander Veterans?
      2. How about experiences among specific Asian American Veteran communities?
      3. What about experiences among specific Pacific Islander Veteran communities?
6. **What cultural norms or values are important for understanding suicide risk and prevention with AAPI Veterans?**
   1. How well do current suicide prevention interventions and resources incorporate these?
   2. To your knowledge, are any current suicide prevention interventions and resources inconsistent with these?
7. **To what extent is suicide discussed among AAPI Veterans? If it is discussed, how is it discussed? Are there any important considerations for how to discuss suicide risk and prevention with AAPI Veterans, including with respect to the specific words or language used?**
8. **We are interested in understanding more about suicide, including risk and protective factors, among AAPI Veterans, including any that may be unique to (or particularly important for) AAPI Veterans. *[Probe throughout: How might these be similar or different to non-AAPI Veterans?]***
   1. In your experience, what factors cause AAPI Veterans to experience suicidal thoughts and behaviors? Which factors cause AAPI Veterans to be at risk for suicide? What factors do you think are important to consider in understanding why some AAPI Veterans die by suicide?
   2. What factors protect against suicide among AAPI Veterans?
   3. How do you think risk and protective factors for suicide differ between Asian American compared to Pacific Islander Veterans? Are there differences that are important for clinicians and researchers to consider?
   4. Similarly, are there important differences within specific Asian American or Pacific Islander groups with respect to suicide risk and protective factors that should be considered?
9. **To your knowledge, what is currently being done to prevent suicide among AAPI Veterans?** [Optional follow-up probe: what is being done to address the risk factors you mentioned earlier?]
   1. How effective are these initiatives for preventing suicide among AAPI Veterans?
   2. What aspects are helpful?
   3. What aspects are not helpful?
   4. What else would help?
   5. To what extent do you think that existing suicide prevention interventions and resources for AAPI Veterans address their needs, values, and cultures? Can you think of ways that such interventions or materials have been adapted?
10. **What else do you think needs to be done to prevent suicide among Asian and Pacific Islander Veterans?**
    1. Where should suicide prevention interventions for Asian and Pacific Islander Veterans be delivered to be most effective? (For example, within VA healthcare settings, community settings, or elsewhere)
    2. Who should deliver such interventions?
    3. What would help with implementing these interventions? What trainings or materials would help?
    4. What could get in the way of providing these interventions?
11. **Are there any important geographical considerations for preventing suicide among AAPI Veterans in your region? For example, barriers to providing suicide prevention interventions or materials in your region? Facilitators?**
12. **[For healthcare providers only] Have you conducted a Safety Plan (sometimes known as a Crisis Response Plan) with AAPI Veterans?** *(If yes, ask the questions below)*
    1. What was that like for you?
    2. Did your experience conducting Safety Plans with AAPI Veterans differ in any way from conducting Safety Plans with Veterans of other racial/ethnic backgrounds?
    3. What did you find to be helpful when conducting Safety Plans with AAPI Veterans?
    4. What did you find to be unhelpful or challenging when conducting Safety Plans with AAPI Veterans?
    5. Did you ever receive any guidance or training on conducting Safety Plans with AAPI Veterans specifically?
    6. Are there any considerations specific to conducting Safety Plans with AAPI Veterans in your region that would be important for us to know about?
    7. What would you recommend or suggest to other healthcare providers who conduct Safety Plans with AAPI Veterans?
    8. What questions do you have related to conducting Safety Plans with your AAPI Veteran patients? What would support you in doing this?
13. **Are you familiar with Caring Contacts?** *(If yes, ask the questions below)*
    1. Have you ever used Caring Contacts or any elements of it with AAPI Veterans? If so, what was that like for you? What did you find to be helpful when using Caring Contacts with AAPI Veterans? What did you find to be unhelpful or challenging when using Caring Contacts with AAPI Veterans?
    2. Did you ever receive any guidance or training on using Caring Contacts with AAPI Veterans specifically?
    3. What considerations do you think would be important for using Caring Contacts with AAPI Veterans? Barriers? Facilitators?
    4. Are there any considerations specific to using Caring Contacts with AAPI Veterans in your region that would be important for us to know about?
    5. Who should deliver the Caring Contact messages when working with AAPI Veterans in your region?
    6. What would you recommend or suggest to other healthcare providers who use Caring Conducts with AAPI Veterans?
    7. What questions do you have related to using Caring Contacts with your AAPI Veteran patients? What would support you in doing this?
14. **How, if at all, should mental health and suicide prevention clinical care differ when working with AAPI Veterans?**
    1. What things do you think providers should be aware of or account for?
    2. What trainings, if any, do you think could be beneficial for those working with AAPI Veterans?
    3. What factors do you think are important to account for during mental health therapy or counseling (e.g., evidence-based psychotherapy, Safety Planning) and suicide risk assessment and prevention with AAPI Veterans?
15. **What resources do you think AAPI Veterans would find most appropriate/useful/worthwhile for suicide prevention?**
    1. What resources do you find helpful when working with AAPI Veterans?
    2. What resources do you wish existed?
    3. In what formats should these be offered?
    4. Who should offer them?
    5. Where should they be offered?
16. **What research is most needed to prevent suicide among AAPI Veterans?**
    1. In your opinion, what is the single most important research question to investigate to help prevent suicide among AAPI Veterans?
    2. What challenges are important to consider in addressing this research question?
    3. What would help in overcoming these challenges?
17. **Is there anything else you think is important to share?**
